# Diagnostic value of [18F]FDG-PET/CT for treatment monitoring in large vessel vasculitis: a systematic review and meta-analysis

**DOI:** 10.1007/s00259-021-05362-8

**Published:** 2021-05-03

**Authors:** K. S. M. van der Geest, G. Treglia, A. W. J. M. Glaudemans, E. Brouwer, M. Sandovici, F. Jamar, O. Gheysens, R. H. J. A. Slart

**Affiliations:** 1 Department of Rheumatology and Clinical Immunology, University of Groningen, University Medical Center Groningen, Hanzeplein 1, 9700RB Groningen, the Netherlands; 2 Clinic of Nuclear Medicine and PET/CT Center, Imaging Institute of Southern Switzerland, Ente Ospedaliero Cantonale, Bellinzona and Lugano, Switzerland; 3 Department of Nuclear Medicine and Molecular Imaging, Lausanne University Hospital and University of Lausanne, Lausanne, Switzerland; 4 Health Technology Assessment Unit, Academic Education, Research and Innovation Area, Ente Ospedaliero Cantonale, Bellinzona, Switzerland; 5 Faculty of Biomedical Sciences, Università della Svizzera italiana, Lugano, Switzerland; 6 Department of Nuclear Medicine and Molecular Imaging, University of Groningen, University Medical Center Groningen, Groningen, the Netherlands; 7 Department of Nuclear Medicine, Cliniques Universitaires Saint-Luc and Institute of Clinical and Experimental Research (IREC), Université Catholique de Louvain (UCLouvain), Brussels, Belgium; 8 Department of Biomedical Photonic Imaging, Faculty of Science and Technology, University of Twente, Enschede, the Netherlands

**Keywords:** Large vessel vasculitis, Giant cell arteritis, Takayasu arteritis, Aortitis, Positron emission tomography computed tomography, Fluorodeoxyglucose F18

## Abstract

**Purpose:**

Monitoring disease activity in patients with large vessel vasculitis (LVV) can be challenging. [18F]FDG-PET/CT is increasingly used to evaluate treatment response in LVV. In this systematic review and meta-analysis, we aimed to summarize the current evidence on the value of [18F]FDG-PET/CT for treatment monitoring in LVV.

**Methods:**

PubMed/MEDLINE and the Cochrane library database were searched from inception through October 21, 2020. Studies containing patients with LVV (i.e. giant cell arteritis, Takayasu arteritis and isolated aortitis) that received treatment and underwent [18F]FDG-PET/CT were included. Screening, full-text review and data extraction were performed by 2 investigators. The risk of bias was examined with the QUADAS-2 tool. Meta-analysis of proportions and diagnostic test accuracy was performed by a random-effects model and bivariate model, respectively.

**Results:**

Twenty-one studies were included in the systematic review, of which 8 studies were eligible for meta-analysis. Arterial [18F]FDG uptake decreased upon clinical remission in longitudinal studies. High heterogeneity (*I*^2^ statistic 94%) precluded meta-analysis of the proportion of patients in which the scan normalized during clinical remission. Meta-analysis of cross-sectional studies indicated that [18F]FDG-PET/CT may detect relapsing/refractory disease with a sensitivity of 77% (95%CI 57–90%) and specificity of 71% (95%CI 47–87%). Substantial heterogeneity was observed among the cross-sectional studies. Both variation in clinical aspects and imaging procedures contributed to the heterogeneity.

**Conclusion:**

Treatment of LVV leads to reduction of arterial [18F]FDG uptake during clinical remission. [18F]FDG-PET/CT has moderate diagnostic accuracy for detecting active LVV. [18F]FDG-PET/CT may aid treatment monitoring in LVV, but its findings should be interpreted in the context of the clinical suspicion of disease activity. This study underlines the relevance of published procedural recommendations for the use of [18F]FDG-PET/CT in LVV.

**Supplementary Information:**

The online version contains supplementary material available at 10.1007/s00259-021-05362-8.

## Introduction

Giant cell arteritis (GCA), Takayasu arteritis (TAK) and isolated (non-infectious) aortitis are the main forms of large vessel vasculitis (LVV) [1, 2]. GCA is frequently associated with cranial symptoms, such as headache and jaw claudication [3], whereas limb claudication and loss of peripheral pulsations are more common in patients with TAK [4]. Constitutional symptoms can be observed in all three forms of LVV. Arterial occlusion in LVV may lead to ischaemic damage of end organs (eye, brain, internal organs), whereas progressive aortic dilatation poses the risk of aortic dissection. The erythrocyte sedimentation rate (ESR) and C-reactive protein (CRP) level are frequently elevated at diagnosis [3]. LVV is typically treated with high-dose glucocorticoids but biological disease-modifying anti-rheumatic drugs are increasingly used in the management of LVV. Anti-interleukin(IL)-6 receptor therapy is effective as maintenance therapy for GCA [5], whereas anti-tumour necrosis factor (TNF)α therapy is widely used in TAK [6]. Monitoring disease activity during treatment can be challenging since none of the clinical symptoms and laboratory markers are entirely specific for LVV. The ESR and CRP levels may remain normal during relapse [7, 8]. Moreover, anti-IL-6 receptor therapy precludes a rise of inflammatory markers by direct interference with the acute phase response. Therefore, additional modalities for the assessment of disease activity are warranted.

Imaging tools are increasingly applied in treatment monitoring of patients with LVV. Ultrasonography of temporal and axillary arteries is recommended as a first-line diagnostic test in patients with suspected GCA [9, 10]. The characteristic halo sign in the temporal arteries appears to gradually disappear upon treatment, whereas this abnormality may persist in the axillary arteries during clinical remission [11]. Magnetic resonance angiography (MRA) and computed tomography angiography (CTA) are extensively used in the diagnostic work-up of GCA, but little is known about their use to monitor treatment response [12]. These imaging modalities are also used for monitoring disease activity in TAK and aortitis, although evidence is even more scarce [13].

Imaging with 2-deoxy-2-[18F]fluoro-d-glucose ([18F]FDG) positron emission tomography combined with low-dose computed tomography ([18F]FDG-PET/CT) is a valuable diagnostic tool in LVV [9, 14]. [18F]FDG accumulates in metabolically active immune cells and stromal cells via the glucose transporter. A growing number of studies have evaluated [18F]FDG-PET/CT during treatment in patients with LVV, but its clinical value remains unclear. In this systematic review and meta-analysis, we aimed to summarize current evidence on the role of [18F]FDG-PET/CT for treatment monitoring in patients with LVV.

## Methods

This study is reported in agreement with the Preferred Reporting Items for a Systematic Review and Meta-Analysis (PRISMA) statement. No ethical approval or informed consent was required.

### Search strategy

A comprehensive search of records through the PubMed/MEDLINE and Cochrane Library databases was carried out (date of the last search: October 21, 2020). Search terms included ‘giant cell arteritis’, ‘Takayasu arteritis’, ‘aortitis’, ‘FDG’, ‘PET’, ‘positron emission tomography’, ‘follow-up’ and ‘response’. A detailed overview of the full search strategy is provided in Supplemental Table 1. The search was restricted to English language articles. In order to achieve a more comprehensive search, the references of the selected articles were screened manually by two investigators (OG, RS).

### Study selection

Two reviewers (KG, GT) independently screened the titles and abstracts. Predefined inclusion criteria were original articles performing [18F]FDG-PET/CT for monitoring treatment response in patients with large vessel vasculitis (i.e. GCA, TAK or non-infectious aortitis). Exclusion criteria were (a) review articles, letters, comments, editorials, study protocols; (b) case reports or small case series (less than 10 patients with data of interest); (c) articles not available in English and (d) articles outside the scope of the current review (e.g. articles reporting [18F]FDG-PET without CT, animal studies, studies applying other tracers than [18F]FDG and studies related to infectious aortitis). The following studies were selected for the meta-analysis: (a) studies reporting sufficient data to evaluate the proportion of patients in which [18F]FDG-PET/CT remained positive during clinical remission following an initially positive [18F]FDG-PET/CT at baseline reflecting active disease; (b) studies reporting sufficient data to evaluate the diagnostic accuracy of [18F]FDG-PET/CT to discriminate between clinical relapse and remission; (c) studies in which at least 90% of patients received treatment at the time of the treatment monitoring scan. In case of potential overlap between studies from the same centre, only data from the largest study was used in the meta-analysis. Disagreements were solved through consensus between the reviewers.

### Data extraction

All data extraction was performed by two independent reviewers. The following data was collected: authors, year of publication, country, study design (prospective, retrospective), LVV population, reference standard for LVV, number of scans, age and sex (FJ, OG); hybrid imaging modality, [18F]FDG injected activity, time interval between [18F]FDG injection and image acquisition, scan coverage, image analysis and definition of positive findings (OG; RS); arterial regions examined (AG, MS); additional study design (longitudinal, cross sectional), disease stage, disease duration, reference standard for disease activity, treatment, main findings related to [18F]FDG uptake during treatment (KG, RS). The authors were not contacted to retrieve unpublished data.

### Quality assessment

The revised ‘Quality Assessment of Diagnostic Accuracy Studies’ tool (QUADAS-2) was used to evaluated the quality of all studies in the systematic review. The latter was used to assess the risk of bias for the following criteria: patient selection, index test, reference test and flow/timing, whereas applicability concerns were assessed for patient selection, index and reference test.

### Statistical analysis

A meta-analysis of the proportion of patients in which the [18F]FDG-PET/CT remained positive during clinical remission was performed by the Stuart-Ord (inverse double arcsine square root) method and a DerSimonian-Laird (random effects) model. Heterogeneity was evaluated and a *I*^2^ statistic > 75% precluded evaluation of the pooled proportion. A bivariate model was used to assess the summary estimates of sensitivity, specificity, diagnostic odds ratio (DOR), positive likelihood ratio (LR+) and negative likelihood ratio (LR−). Pooled data were given with 95% confidence intervals (95% CI) and displayed using forest plots and hierarchical summary receiver operating characteristics (HSROC) plots. Likelihood ratios of more than 2.00 or less than 0.50 with 95% CI not including 1.00 were considered statistically significant. Publication bias was evaluated with an effective sample size (ESS) funnel plot and the associated regression test of asymmetry [15]. A threshold effect was evaluated: (a) by comparing the sensitivity and specificity as determined by the bivariate model to those obtained by a univariate random-effects model (DerSimonian-Laird method) and (b) by evaluating Spearman’s correlation coefficient of the logit of sensitivity and logit of 1-specifity. Proportion meta-analysis and the associated *I*^2^ statistic were evaluated with StatsDirect 3.2.109. Bivariate model analysis, HSROC plot and evaluation of funnel plot asymmetry were performed with STATA version 15.1 (*metandi* and *midas* commands). Forest plots were constructed in Review Manager version 5.3 and StatsDirect 3.2.109. Assessment of the threshold effect was performed with MetaDiSc 1.4. No additional sub-analyses were performed.

## Results

### Literature search

**Fig. 1:**
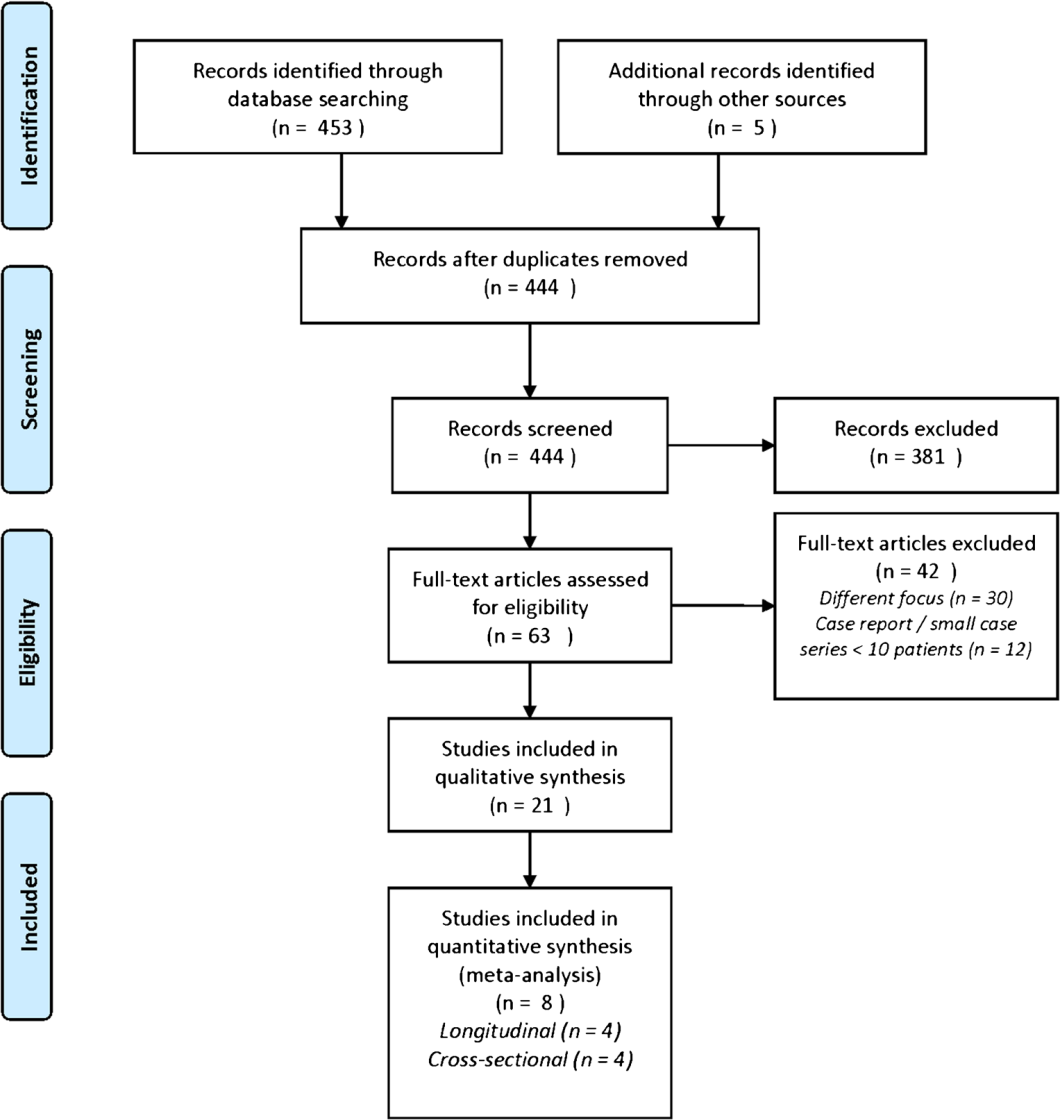
PRISMA flow diagram

A comprehensive database search yielded a total of 444 unique records (Fig. 1). The earliest reference is dated from July 1987. Title and abstract screening led to exclusion of 381 records. A further 42 articles were excluded following full-text assessment. Eventually, 21 studies were selected for the qualitative analysis (systematic review) [16, 17, 18–36]. Eight studies were included in the meta-analysis since these reports contained sufficient data to either evaluate the accuracy of [18F]FDG-PET/CT for discriminating between active disease and remission during treatment [19, 23, 25, 33] or to evaluate the proportion of patients in which the scan normalized during clinical remission in patients on treatment [17, 20, 22, 36].

### Qualitative analysis (systematic review)

#### Study and patient characteristics

Table 1 summarizes the main characteristics of the 21 included studies. All articles have been published in the last decade with 16 (76%) being published in the past 5 years. Eleven studies (51%) were performed in Europe, 5 studies (24%) in Asia, 4 studies (19%) in the USA and 1 study in Australia. Studies with a retro- or prospective design were equally distributed (10 studies each), whilst one study consisted of a retrospective subgroup analysis of a large European prospective trial. Eleven studies (52%) reported data on serial [18F]FDG-PET/CT scans in patients with LVV, 8 studies (38%) reported cross-sectional data, whereas 2 studies (10%) contained both longitudinal and cross-sectional data. The vast majority of studies used the ACR criteria 1990 for GCA or TAK as a reference standard for LVV [37, 38]. All studies reporting gender distribution and age showed a female predominance for both vasculitis types and an overall lower mean/median age for patients with TA compared to patients with GCA. However, several studies reported a mean/median age > 40 years for patients with TA.

**Table 1:** Study and patient characteristics

Authors	Country	Study design		Type of LVV	Reference standard for LVV diagnosis	No. of [18F]FDG-PET/CT scans (no. of patients)	Median or * mean age (yrs)	% male	CRP/ESR available
Alibaz-Oner et al. (2015)	Turkey	Prospective	Cross sectional	TAK	ACR criteria 1990	14 (14)	39*	21	No
Banerjee et al. (2020)	USA	Prospective	Longitudinal	TAK, GCA	ACR criteria 1990 or modifiedcriteria for GCA	156 (52; 21 TAK and 31 GCA)	30 (TAK); 72 (GCA)	24 (TAK); 26 (GCA)	Yes
Bruls et al. (2016)	Belgium	Prospective	Longitudinal	TAK, GCA, aortitis	Clinical, biochemical, biopsyand PET/CT results + ACRcriteria 1990 (*n* = 4)	45 (15; 2 TAK, 4 GCA, 6 aortitis, 3 other)	65* total population	44 total population	No
Castellani et al. (2016)	Italy	Retrospective	Cross sectional and longitudinal	TAK, GCA	Not specified	41 (21; 5 TAK and 16 GCA)	61* TAK; 70* GCA	33 total population; 33 (TAK); 22 (GCA)	Yes
de Boysson et al. (2017)	France	Retrospective	Longitudinal	GCA	ACR criteria 1990 or 2 ACRcriteria in combinationwith PET/CT positivity	59 (25)	69	32	Yes
Grayson et al. (2018)	USA	Prospective	Cross sectional	TAK, GCA	ACR criteria 1990 or modified criteria for GCA	115 (56; 26 TAK and 30 GCA)	31* TAK, 69* GCA	31 (TAK); 30 (GCA)	Yes
Henes et al. (2011)	Germany	Retrospective	Longitudinal	TAK, GCA	Clinical	36 (10; 4 TAK and 6 GCA)	48 (TAK); 63 (GCA)	20	Yes
Incerti et al. (2017)	Italy	Retrospective	Cross sectional	TAK	ACR criteria 1990	30 (30)	43	13	Yes
Lee et al. (2012)	South Korea	Retrospective	Longitudinal	TAK	ACR criteria 1990 (CTA or MRAinstead of conventional angiography)	53 (38) with 13 (13) used in systematic review	43*	11	Yes
Li et al. (2019)	China	Prospective	Cross sectional	TAK	ACR criteria 1990 (CTA or MRAinstead of conventional angiography)	22 (71)	34* total population; 34* PET-CT group	16 total population; 23 PET-CT group	Yes
Martinez-Rodriguez et al. (2018)	Spain	Retrospective	Longitudinal	GCA, aortitis	ACR criteria 1990	74 (37)	67*	24	Yes
Nielsen et al. (2018)	Denmark	Prospective	Longitudinal	GCA	Clinical, biochemical, biopsy,FDG results and ACR criteria 1990	48 (24)	69*	33	Yes
Park et al. (2018)	South Korea	Prospective	Longitudinal	TAK	ACR criteria 1990	22 (11)	47*	0	Yes
Quinn et al. (2018)	USA	Prospective	Cross sectional	TAK, GCA	ACR criteria 1990 or modifiedcriteria for GCA	114 (65; 30 TAK and 35 GCA)	33* TAK; 68* GCA	33 (TAK); 20 (GCA)	Yes
Regola et al. (2020)	Italy	Retrospective	Longitudinal	GCA	ACR criteria 1990 or biopsyor FDG results	30 (32) with 22 (11) used in a systematic review	74 total population; PET-CT group not specified	22 total population; r PET-CT group not specified	Yes
Rimland et al. (2020)	USA	Prospective	Cross sectional and longitudinal	TAK, GCA	ACR criteria 1990 ormodified criteria for GCA	240 (112; 56 TAK and 56 GCA)	34 (TAK); 71 (GCA)	20 (TAK); 21 (GCA)	Yes
Sammel et al. (2020)	Australia	Prospective	Longitudinal	GCA	Biopsy and clinical follow-up for > 6 months	36 (21) with 30 (15) included in systematic review	73 total population; 71 for dual time-point	33	Yes
Santhosh et al. (2014)	India	Retrospective	Cross sectional	TAK	ACR criteria 1990	60 (51) with 43 (38) included in systematic review	Total population not reported; 30* patients at diagnosis	25	No
Schramm et al. (2019)	Germany	Retrospective	Cross sectional	TAK, GCA	ACR criteria 1990 and imaging	80 (62) including 52 FDG-PET/CT scans without CT	Not reported	Not reported	Yes
Tezuka et al. (2012)	Japan	Retrospective	Cross sectional	TAK	ACR criteria 1990 and guideline for the management of vasculitissyndrome (Japanese circulationsociety 2008)	39 (39) with 29 (29) included in a systematic review	30	10	Yes
Vitiello et al. (2018)	Italy	Retrospective	Longitudinal	GCA	ACR criteria 1990	24 (12)	69*	33	Yes

*GCA*, giant cell arteritis; *LVV*, large vessel vasculitis; *TAK*, Takayasu arteritis; * mean age is provided rather than median age.

* mean age is provided rather than median age

#### Technical aspects

The technical aspects of [18F]FDG-PET/CT in the 21 studies are summarized in Table 2. [18F]FDG-PET scanning was performed together with low-dose CT except for one study in which part of the scans (65%) was performed without CT [34]. Contrast-enhanced CT was performed in two studies. Four studies from the same centre reported that [18F]FDG-PET/MRI was applied in paediatric patients, whereas the adult patients underwent [18F]FDG-PET/CT [16, 21, 29, 31]. The injected [18F]FDG activity was quite heterogeneous and included both weight-based and fixed activities. The [18F]FDG uptake time was 60 min in 13 studies (62%), < 60 min in 2 studies (10%) and 120–180 min in 4 studies (19%). Two studies (10%) did not report this technical aspect. The vast majority of scans covered the skull (either from the vertex or skull base) to the thigh region, including the (middle)large arteries, whilst some studies also included the feet. Reconstruction algorithms or adherence to EARL was not always specified. [18F]FDG-PET/CT image interpretation was primarily performed by visual analysis in 11 studies (52%) and a combination of visual analysis and semi-quantitative analysis using the maximum standardized uptake value (SUV) in 7 studies (33%). In 8 studies (38%), a target-to-background ratio (TBR) was used: 5 studies (24%) applied a target-to-liver ratio and 5 studies (24%) used the blood pool activity as background. The definition of positive [18F]FDG uptake was different among the included studies, but the majority of studies used the liver as the reference organ. In 6 studies (29%), visual uptake equal or higher to the liver was considered positive whilst uptake higher than the liver (either visual or semi-quantitatively) was defined as positive in 3 studies (14%). One study (5%) used different cutoff points for visual uptake at different arterial regions [33]. Six studies (29%) did not report any specific definition of positive [18F]FDG-PET/CT finding.

**Table 2:** [18F]FDG-PET/CT characteristics in the studies

Study	[18F]FDG Imaging modality	Injected activity	Interval [18F]FDG injection-image acquisition	Scan coverage	Arterial regions examined	Image analysis	Definition of positive [18F]FDG-PET/CT finding
Alibaz-Oner et al. (2015)	PET/CT (low-dose CT)	3.7 MBq/kg	60 min	Mid-skull to mid-thigh	AoAsc, AoArch, AoDesc, AoAbd, Innom, CarotCom, Subclav, Renal	Visual^a^	Visual ≥ 2^a^
Banerjee et al. (2020)	PET/CT (low-dose CT) *Paediatrics: PET/MRI*	370 MBq *Paediatrics: 3.7 MBq/kg*	120 min for PET/CT 60 min for PET/MR	Vertex to the proximal thigh	AoAsc, AoArch, AoDesc, AoAbd, Innom, Carot Subclav	1) Visual 2) PETVAS^a^	Visual pattern consistent with vasculitis
Bruls et al. (2016)	PET/CT (low-dose CT, followed by arterial phase CT	3.7 MBq/kg	60 min	Skull to upper-thigh	‘Supra-aortic’, AoThor, AoAbd, Iliac	Visual	Any focal uptake higher than background
Castellani et al. (2016)	PET/CT (low-dose CT)	199–478 MBq	50–60 min	Mid-skull to distal lower extremities	‘Supra-aortic’, AoThor, AoAbd, Iliofem	1) Visual 2) Total visual score 11 aortic tree regions 3) TBR (SUVmean artery/SUVmean liver) 4) Mean TBR 11 aortic tree regions	Calculated cutoff 12 for total visual score and 0.653 for mean TBR 11 aortic tree regions
De Boysson et al. (2017)	PET/CT (low-dose CT)	4 MBq/kg	60 ± 5 min	Whole body	AoThor, AoAbd, Carot, Subclav, Axil, Iliofem, ‘upper and lower limb arteries’	Visual^a^	Visual = 3^a^
Grayson et al. (2018)	PET/CT(low-dose CT) *Paediatrics: PET/MRI*	370 MBq *Paediatrics: 3.7 MBq/kg*	120 min	Vertex to mid-thigh	AoAsc, AoArch, AoDesc, AoAbd, Innom, Carot, Subclav, Axil, Iliac, Fem	1) Nuclear medicine physician global assessment 2) Visual 3) PETVAS^a^	Visual pattern consistent with vasculitis
Henes et al. (2011)	PET/CT (low-dose CT)	Not reported	60 min	Mid-skull to knee/mid-thigh (presumably)	AoAsc/AoArch, AoDesc, AoAbd, CarotCom, Subclav/Axil, IliacCom, FemSup	Visual^a^	Visual ≥ 2^a^
Incerti et al. (2017)	PET/CT (low-dose CT)	370 MBq	60 min	Vertex to mid-thigh	AoAsc, AoArch, AoDesc, AoAbd, Innom, CarotCom, Subclav, Axil, Humeral, Renal, IliacCom, Fem, Popliteal	1) Visual 2) SUVmax	Visual uptake equal to or higher than liver
Lee et al. (2012)	PET/CT(low-dose CT)	370 MBq	40–60 min	Mid-skull to mid-thigh	AoAsc, AoArch, AoDesc, AoAbd, Innom, CarotCom, Subclav, IliacCom	1) Visual^a^ 2) TBR (SUVmax artery/SUVmax liver)	Visual ≥ 2^a^
Li et al. (2019)	PET/CT (low-dose CT)	37 MBq/13 kg	60 min	Only carotid artery	CarotCom	Visual^a^	Visual ≥ 2^a^
Martínez-Rodríguez et al. (2018)	PET/CT (low-dose CT)	7 MBq/kg	180 min	Whole body	‘Entire aortic wall’	TBR (SUVmax artery/SUVmax blood pool)	Not reported for the relevant disease monitoring scans
Nielsen et al. (2018)	PET/CT (low-dose CT)	Baseline scan: 5 MBq/kg Second scan: 200 MBq	60 min	Baseline scan: Skull to mid-thigh Second scan: Limited field of view (minimum neck and thorax)	AoAsc, AoArch, AoDesc, Carot, SubclavAsc, SubclavDesc, Axil, Vertebral	1) Visual^a^ 2) TBR (SUVmax artery/SUVmean venous)	Visual ≥ 3^a^
Park et al. (2018)	PET/CT (low-dose CT)	5.18 MBq/kg	60 min	Skull to proximal thigh or whole-body	AoAsc, AoArch AoDesc, AoAbd., Innom, CarotCom, Subclav, Iliac	1) Visual^a^ 2) PETVAS 3) SUVmax 4) TBR (SUVmax artery/SUVmean vein) 5) TBR (SUVmax artery/SUVmean liver)	Visual ≥ 2^a^
Quinn et al. (2018)	PET/CT (low-dose CT) *Paediatrics: PET/MRI*	370 MBq (presumably)	Not reported	Not reported	AoAsc, AoArch, AoDesc, AoAbd, Innom, Carot, Subclav, Axil, Iliac, Fem	1) Visual 2) PETVAS^a^	Visual uptake higher than liver
Regola et al., (2020)	PET/CT (low-dose CT)	Not reported	Not reported	Not reported	AoAsc, AoArch, AoDesc, AoAbd, Innom, Carot, Subclav	1) PETVAS^a^ 2) TBR (SUVmax artery/SUVmax liver)	Not reported for the relevant disease monitoring scans
Rimland et al. (2020)	PET/CT (low-dose CT) *Paediatrics: PET/MRI*	370 MBq	120 min	Vertex to mid-thigh	AoAsc, AoArch, AoDesc, AoAbd, Innom, Carot, Subclav	PETVAS^a^	Not reported for the relevant disease monitoring scans
Sammel et al. (2020)	PET/CT (low-dose CT)	100 MBq	60 min	Vertex to diaphragm (head/neck/horax)	AoAsc, AoArch, AoDesc, Innom, Carot, Subclav, Axil, Temporal, Occipital, Maxillary, Vertebral	Total vascular score 18 arterial regions ^b^	Not reported for the relevant disease monitoring scans
Santhosh et al. (2014)	PET/CT (low-dose CT)	370 MBq	60 min	Skull base to mid-thigh	AoAsc, AoArch, AoDesc, AoAbd, Innom, CarotCom, Subclav, Brachial, Pulmonary, Superior mesenteric, IliacCom	Visual	Visual = 3 in ascending aorta, visual ≥ 2 aortic arch, any uptake descending/abdominal aorta^a^
Schramm et al. (2019)	PET/CT (low-dose CT or contrast-enhanced CT) PET without CT	3 MBq/kg	60 min	Skull base to proximal thigh	AoAsc, AoArch, AoDesc, AoAbd	TBR (SUVmax artery/SUVmean of the liver, inferior vena cava, superior vena cava or right atrium)	Not reported for the relevant disease monitoring scans
Tezuka et al. (2012)	PET/CT (low-dose CT)	3.7 MBq/kg	60 min	Vertex to mid-thigh	AoAsc, AoArch, AoDesc, Innom, Carot, Subclav, Renal	1) SUVmax artery 2) TBR (SUVmax artery/SUVmean inferior vena cava)	Not reported for the relevant disease monitoring scans
Vitiello et al. (2018)	PET/CT (low-dose CT)	3.7 MBq/kg	60 min	Vertex to mid-thigh (presumably)	AoThor, AoAbd, Carot, Subclav, Iliac, Fem	1) Visual 2) SUVmean	Visual pattern consistent with vasculitis

Information on image analysis is focussed on data relevant to treatment monitoring as reported in Table 3 and Supplemental Table 2. *AoAbd*, abdominal aorta; *AoArch*, aortic arch; *AoAsc*, ascending aorta; *AoDesc*, descending aorta; *AoThor*, thoracic aorta; *Axil*, axillary artery; *Carot*, carotid artery; *CarotCom*, common carotid artery; *Fem*, femoral artery; *IliacCom*, common iliac artery; *Iliofem*, iliofemoral artery; *Innom*, innominate (brachiocephalic) artery; *PETVAS*, PET vascular activity score (sum of visual scores in different arterial regions). *Subclav*, subclavian artery; *SubclavAsc*, ascending subclavian artery; *SubclavDesc*, descending subclavian artery; *TBR*, target to background ratio

^a^Visual 0, no uptake; visual 1, uptake lower than liver; visual 2, FDG uptake equal to the liver; visual 3, FDG uptake more than liver present

^b^Visual 0, no uptake; visual 1, minimal/equivocally increased uptake; visual 2, moderate/clearly increased uptake; visual 3, very marked uptake (no comparison to the liver was made)

#### Methodological quality of studies

**Fig. 2:**
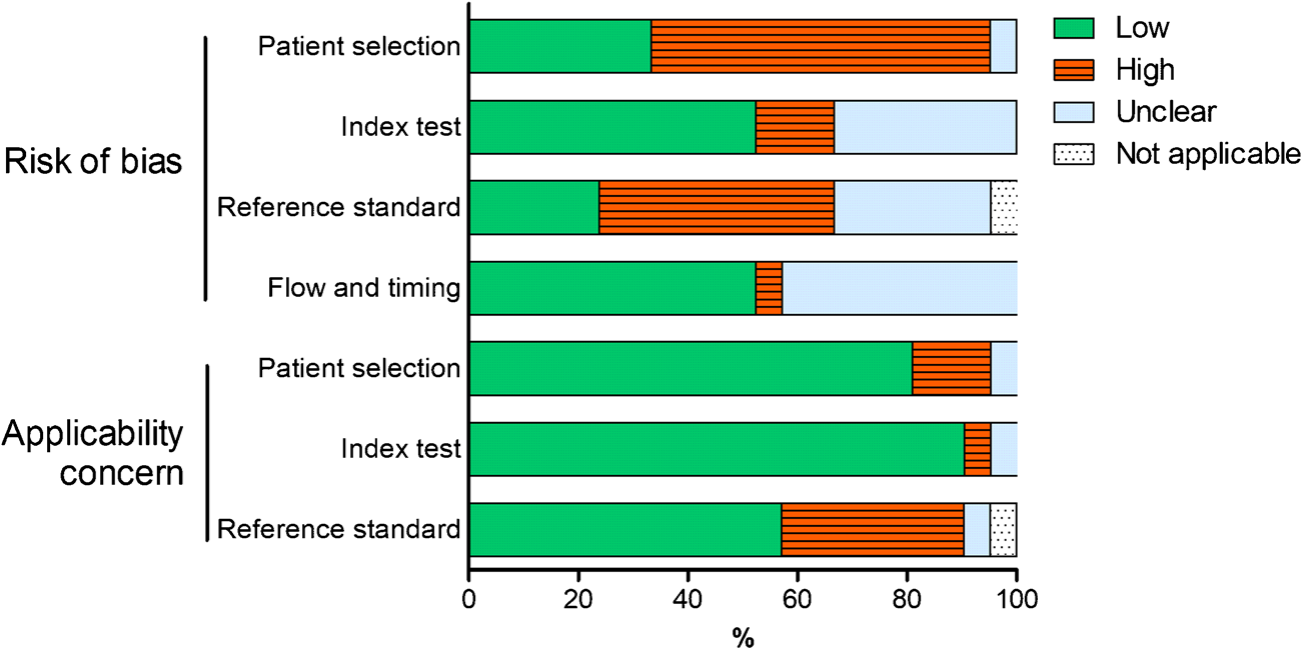
Overall summary of QUADAS-2 items. Risk of bias and concern of applicability was assessed for 21 studies in the systematic review

Patient selection and the reference standard were the main sources of bias in the 21 studies (Fig. 2 and Supplemental Figure 1). Concerns regarding the applicability of the findings were related to the reference standard in studies applying instruments (i.e. NIH criteria, BVAS or ITAS2010) that have not been thoroughly validated for treatment monitoring of patients with LVV [12, 39].

#### Main findings of qualitative assessment

Five longitudinal studies (24%) only contained patients with newly diagnosed LVV, whereas the other longitudinal studies also included patients with relapsing and/or refractory disease (Supplemental Table 2). The median disease duration in the latter studies ranged from 6 months to 4.8 years. The cross-sectional studies mostly contained patients on treatment with a disease duration ranging from 2.0–6.9 years (Table 3). Four cross-sectional studies (19%) also contained patients with newly diagnosed LVV: in 1 study, at least 90% of all scans were performed during treatment [34], whereas this was unclear (at least 54%) in the other 3 studies [21, 29, 31]. Clinical disease activity was determined according to standardized instruments (i.e. NIH criteria, BVAS or ITAS2010) in 6 studies (29%). Physician’s clinical assessment (i.e. symptoms, physical signs, with/without inflammation markers) was used as the reference standard for disease activity in the other 15 studies (71%). [18F]FDG-PET/CT findings were involved in the reference standard for disease activity in 2 studies (10%). Treatment included glucocorticoid therapy, conventional synthetic disease-modifying anti-rheumatic drugs (DMARDs) and/or biological DMARDs. Several studies investigated a specific DMARD: anti-IL-6R therapy (i.e. tocilizumab) (*n* = 3) [16, 30, 36], anti-TNFα therapy (i.e. infliximab) (*n* = 2) [16, 28] and cyclophosphamide (*n* = 1) [22].

**Table 3:** Main findings in cross-sectional studies on hybrid imaging for monitoring of treatment response

Study	No. of patients and disease stage	Disease duration since diagnosis	Assessment of disease activity	Treatment during scan	FDG uptake during treatment
Alibaz-Oner et al. (2015)^a^	TAK (*n* = 14) *Unclear*	Mean 5.7 years (SD 5)	Physician’s global assessment	Oral methylprednisolone (*n* = 13), AZA (*n* = 7), MTX (*n* = 4), LEFL (*n* = 2), anti-TNF (*n* = 3)	Scan in patients with *persistent acute phase response without signs/symptoms of clinically active disease* (*n* = 14) • Vascular FDG uptake grade ≥ 2† found in 9/14 patients • Number of arteries with FDG uptake grade ≥ 2, median 2 (range 1–5)
Castellani et al. (2016)	TOTAL (*N* = 21) GCA (*n* = 16) TAK (*n* = 5) *Refractory* *Remission* *Relapse*	Unclear	Assessment of clinical course (clinical and laboratory data, response to GC treatment); no standardized criteria	GC treatment or immunosuppressants	Diagnostic accuracy of scan for assessment of disease activity (*n* = 41 scans; *clinically active disease* during 15 scans; *clinical remission* during 26 scans): • Visual grading: supra-aortic branches AUC 0.687, Sens 73%, Spec 54%; thoracic aorta AUC 0.744, Sens 67%, Spec 73%; abdominal aorta AUC 0.692, Sens 80%, Spec 68%; iliofemoral arteries AUC 0.686, Sens 33%, Spec 96%; total visual score of 11 regions in aortic tree AUC 0.736, Sens 73%, Spec 81% • TBR (SUVmean artery/SUVmean liver): supra-aortic branches AUC 0.810, Sens 93%, Spec 58%; thoracic aorta AUC 0.777, Sens 80%, Spec 65%; abdominal aorta AUC 0.738, Sens 93%, Spec 58%; iliofemoral arteries AUC 0.821, Sens 87%, Spec 81%; in entire aortic tree AUC 0.827, Sens 93%, Spec 62%
Grayson et al. (2018)^b^	TOTAL (*n* = 56) GCA (*n* = 30) TAK (*n* = 26) *Newly diagnosed* *Remission* *Relapse* *Possibly refractory* *At least 69/111 (62%) scans during treatment*	Mean 6.9 years (SD 8.9)	Active disease = presence of clinical feature attributed to vasculitis (fatigue or elevated acute phase reactants alone not sufficient) Remission = absence of clinical feature attributed to vasculitis	Clinical active disease (40 scans): prednisone used during 24/40 scans; immune medication used during 27/40 scans Clinical remission (71 scans); prednisone used during 42/71 scans; immune medication used during 42/71 scans	Scan during clinically active disease (*n* = 40 scans): nuclear medicine physician global impression of scan consistent with vasculitis in 34/40 scans Scan during clinical remission (*n* = 71 scans): nuclear medicine physician global impression of scan consistent with vasculitis in 41/71 scans Diagnostic accuracy of scan for assessment of disease activity: • Nuclear medicine physician global impression, Sens 85%, Spec 42% • PETVAS, AUC 0.72, OPC 20, Sens 68%, Spec 71%
Incerti et al. (2017)	TAK (*n* = 30) *Remission* *Relapse* *Possibly refractory*	Median 5 years (range 0–17)	NIH criteria	Any immunosuppressive treatment (*n* = 27): GC treatment (*n* = 24) with median dose 5 mg (range 4–50 mg), MTX (*n* = 13), AZA(*n* = 6), MMF (*n* = 1), LEFL (*n* = 1), sirolimus (*n* = 1), sulfasalazine (*n* = 1), IFX (*n* = 5), adalimumab (*n* = 2), TCZ (*n* = 2), golimumab (*n* = 1)	Scan during *clinically active disease* (*n* = 18) • Vascular FDG uptake grade 1 (= FDG uptake equal to/higher than liver) in 9/18 patients, and in 6/18 patients if FDG uptake at vascular graft is excluded • Number of lesions with significant FDG uptake grade 1, median 0 (0–8), and median 0 (range 0–8) if FDG uptake at vascular graft is excluded • SUVmax, median 1.4 (range 0.1–6.7), and median 0.1 (range 0.1–6.2) if FDG uptake at vascular graft is excluded Scan during *clinical remission* (*n* = 12) • Vascular FDG uptake grade 1 in 7/12 patients, and in 7/12 patients if FDG uptake at vascular graft is excluded • Number of lesions with significant FDG uptake grade 1, median 1 (range 0–8), and median 1 (range 0–6) if FDG uptake at vascular graft is excluded • SUVmax, median 2.8 (range 0.1–9.8), and median 2.8 (range 0.1–9.8) if FDG uptake at vascular graft is excluded *NB 7/30 patients received a total of 11 arterial grafts; FDG-PET/CT performed after median 37 months (range 12–55 months) after surgery showed vascular FDG uptake grade 1 at 10/11 arterial grafts. SUVmax at grafts, median 3.9 (range 0.1–6.7).*
Li et al. (2019)	TAK (*n* = 22) *Remission* *Relapse* *At least 69/71 (97%) patients on treatment*§	Mean 5.4 years (SD 5.5)§	ITAS2010	Treatment of all patients in the study (*n* = 71) including all patients that underwent FDG-PET/CT (*n* = 22): Prednisone 69/71 patients, CYC 25/71 patients, LEFL13/71 patients, MMF 11/71 patients, MTX 18/71 patients, TCZ 11/71 patients, tacrolimus 2/71 patients, cyclosporine 2/71 patients§	Scan during *clinically active disease* (*n* = 12): Vascular FDG uptake grade ≥ 2† in the carotid artery of 9/12 patients Scan during *clinical remission* (*n* = 10): vascular FDG uptake grade ≥ 2 in the carotid artery of 2/10 patients
Rimland et al. (2020)^b^	TOTAL (*n* = 112) GCA (*n* = 56) TAK (*n* = 56) *Newly diagnosed* *Remission* *Relapse* *Possibly refractory* *At least 61/112 (54%) patients on treatment*	Median 2.4 years (IQR 0.7–8.3)	Physician global assessment on a scale of 0 (remission) to 10 (very active diseases)	Treatment (n = 112) • Prednisone, median dose 5 mg (IQR 0–19.4) • Other immunosuppressant 61/112 patients	Scan during *clinically active disease* (*n* = 82 scans): PETVAS, median 21.5 (IQR 16.8–25.0) (*n* = 82 scans) Scan during *clinical remission* (*n* = 158 scans): PETVAS, median 17.0 (IQR 11.0–21.0) (158 scans)
Santhosh et al. (2014)	TAK (*n* = 38) *Relapse* *Remission*	Mean 2.9 years (SD 0.6)	National Institute of Health criteria and/or positive FDG-PET/CT	‘Immunosuppression’	Scan during *clinically active disease* (*n* = 12 scans) • Pathologic vascular FDG uptake (i.e. grade 3 at ascending aorta, grade ≥ 2 at the aortic arch or large aortic branch, or grade ≥ 1 at descending or abdominal aorta†) in 10 scans Scan during *clinical remission* (*n* = 31 scans) • Pathologic vascular FDG uptake (i.e. grade 3 at ascending aorta, grade ≥ 2 at the aortic arch or large aortic branch, or grade ≥ 1 at descending or abdominal aorta†) in 3 scans Diagnostic accuracy of scan for assessment of disease activity: • Sens 83% and Spec 90%
Schramm et al. (2019)^c,d^	TOTAL (*n* = 62) GCA (*n* =?) TAK (*n* =?) *Newly diagnosed* *Remission* *Relapse* *Possibly refractory* *At least 74/80 (93%) scans on treatment*	Mean 2.0 years (SD 3.3; range 0–15.1)	Physician global assessment based on clinical symptoms and acute phase reactants	• Prednisolone used during 74/80 scans, mean dose 54 mg (SD 113) • Conventional immunosuppressive treatment during 20/80 scans • Biological immunosuppressive treatment during 8/80 scans	Scan during clinically active disease: • TBR (SUVmax aorta/SUVmean liver), mean 1.74 (SD 0.60)^a^ • TBR (SUVmax aorta/SUVmean inferior vena cava), mean 2.76 (SD 1.00)^b^ • TBR (SUVmax aorta/SUVmean superior vena cava), mean 2.66 (SD 1.07)^b^ • TBR (SUVmax aorta/SUVmean right atrium), mean 1.81 (SD 0.4)^b^ Scan during clinical remission: • TBR (SUVmax aorta/SUVmean liver), mean 1.18 (SD 1.26)^a^ • TBR (SUVmax aorta/SUVmean inferior vena cava), mean 1.84 (SD 0.27)^b^ • TBR (SUVmax aorta/SUVmean superior vena cava), mean 1.68 (SD 0.31)^b^ • TBR (SUVmax aorta/SUVmean right atrium), mean 1.79 (SD 0.35)^b^ Diagnostic accuracy of scan for assessment of disease activity: • TBR (SUVmax aorta/SUVmean liver), AUC 0.90, Sens 84%, Spec 83%^a^ • TBR (SUVmax aorta/SUVmean inferior vena cava), AUC 0.84, Sens 75%, Spec 100%^b^ • TBR (SUVmax aorta/SUVmean superior vena cava), AUC 0.88, Sens 92%, Spec 75%^b^ • TBR (SUVmax aorta/SUVmean right atrium), AUC 0.52, Sens 75%, Spec 50%^b^ ^*a*^*Including 28 FDG-PET/CT scans and 52 FDG-PET scans without CT; active disease during 57/80 scans;* ^*b*^*including 28 FDG-PET/CT scans; number of scans with active/inactive disease during scan unclear*
Tezuka et al. (2012)^d^	TAK (*n* = 29) *Relapse* *Remission* (‘stable’)	Unclear	National Institute of Health Criteria	Prednisolone dose, median 10 mg (IQR 6–16) in relapsing patients (*n* = 17) and 8 mg (IQR 2–15) in patients in remission (*n* = 12), and additional immunosuppressant in 5 relapsing patients and 3 patients in remission; i.e. CYC (*n* = 1), cyclosporin (*n* = 3), MTX (*n* = 2) or AZA (*n* = 2)	Scan during clinically active disease (*n* = 17): SUVmax, median 2.6 Scan during clinical remission (*n* = 12): SUVmax, median 1.9 Diagnostic accuracy of scan for assessment of disease activity: • SUVmax, AUC 94% • TBR (SUVmax artery/SUVmean inferior vena cava), AUC 92%
Quinn et al. (2018)^b^	TOTAL (*n* = 65) GCA (*n* = 35) TAK (*n* = 30) *Newly diagnosed* *Remission* *Relapse* *Possibly refractory* *At least 68/114 (60%) scans during treatment*	Median 2.2 years (IQR 0.9–5.2) in patients with scan during active disease, and median 2.8 years (IQR 1.4–7.3) in patients with scan during remission.	Active disease = presence of clinical feature attributed to vasculitis (fatigue or elevated acute phase reactants alone not sufficient) Remission = absence of clinical feature attributed to vasculitis (regardless of acute-phase reactants)	Clinical active disease (*n* = 45 scans): • Prednisone, median dose 5 mg (IQR 0–30) • Immune medications used during 28/45 scans Clinical remission (*n* = 69 scans): • Prednisone, median dose 5 mg (IQR 0–10) • Immune medications used during 40/69 scans	Scan during clinically active disease (*n* = 45 scans) • Vascular FDG uptake higher than liver in 37/45 scans • PETVAS, median 20.5 (IQR 14–25) Scan during clinical remission (*n* = 69 scans) • Vascular FDG uptake higher than liver in 43/69 scans • PETVAS, median 18 (IQR 14–25)

Glucocorticoid treatment was used orally unless stated otherwise. *AZA*, azathioprine; *CYC*, cyclophosphamide; *GC*, glucocorticoid; *GCA*, giant cell arteritis; *IFX*, infliximab; *ITAS2010*, Indian Takayasu’s Arteritis Activity Score 2010; *IQR*, interquartile range; *IV*, intravenous; *LEFL*, leflunomide; *MMF*, mycophenolate (mofetil); *MTX*, methotrexate; *n*, number of patients (unless stated otherwise); *NIH*, National Institute of Health; *SD*, standard deviation; *TAK*, Takayasu arteritis; *TBR*, target to background ratio; *TCZ*, tocilizumab

†Vascular FDG uptake grading system: 0 = no uptake, 1 = less than liver, 2 = equal to the liver, 3 = more than liver

§Including data obtained from patients without relevant data

^a^Not included in the meta-analysis: uncertainty about disease activity during scan

^b^Not included in the meta-analysis: uncertain if at least 90% of patients were on treatment

^c^Not included in the meta-analysis: part of scans were [18F]FDG-PET without CT

^d^Not included in the meta-analysis: no data on the number of true positives, false positives, false negatives and true negatives

**Fig. 3:**
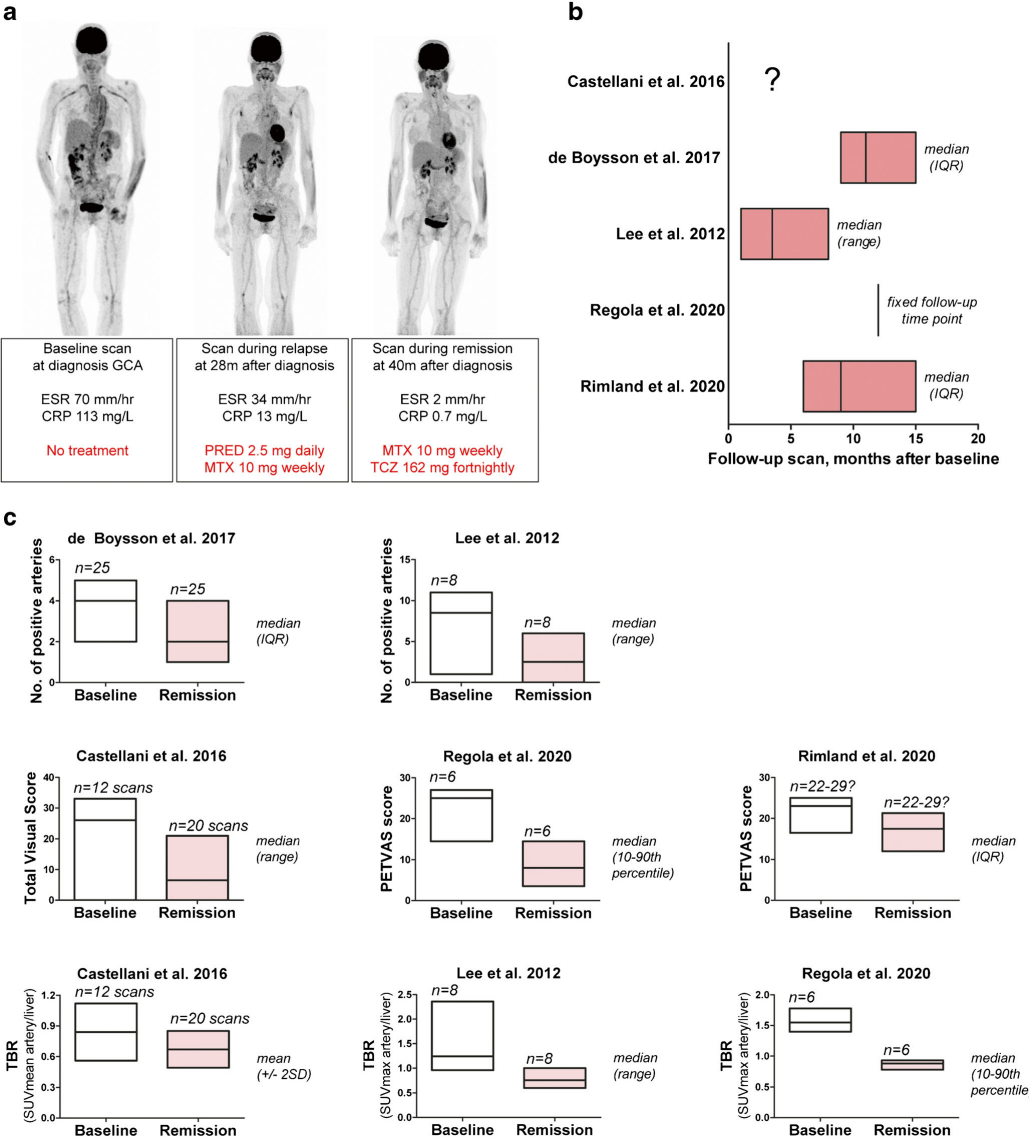
Modulation of quantitative [18F]FDG-PET/CT measures upon clinical remission in longitudinal studies. Per scan data or per patient data at baseline and during serial scans were obtained, if the disease activity during the scans was clearly defined in the studies. **a** Representative [18F]FDG-PET/CT scans of a patients with giant cell arteritis (GCA). Scans were performed at diagnosis and during immunosuppressive treatment. **b** Timing of follow-up scans and **c** quantitative PET measures (including no. of positive arteries, composite PET scores, target to background ratio (TBRs) in the included, longitudinal studies. CRP, C-reactive protein; ESR, erythrocyte sedimentation rate; MTX, methotrexate; PRED, prednisolone; TCZ, tocilizumab (anti-IL-6 receptor therapy)

**Fig. 4:**
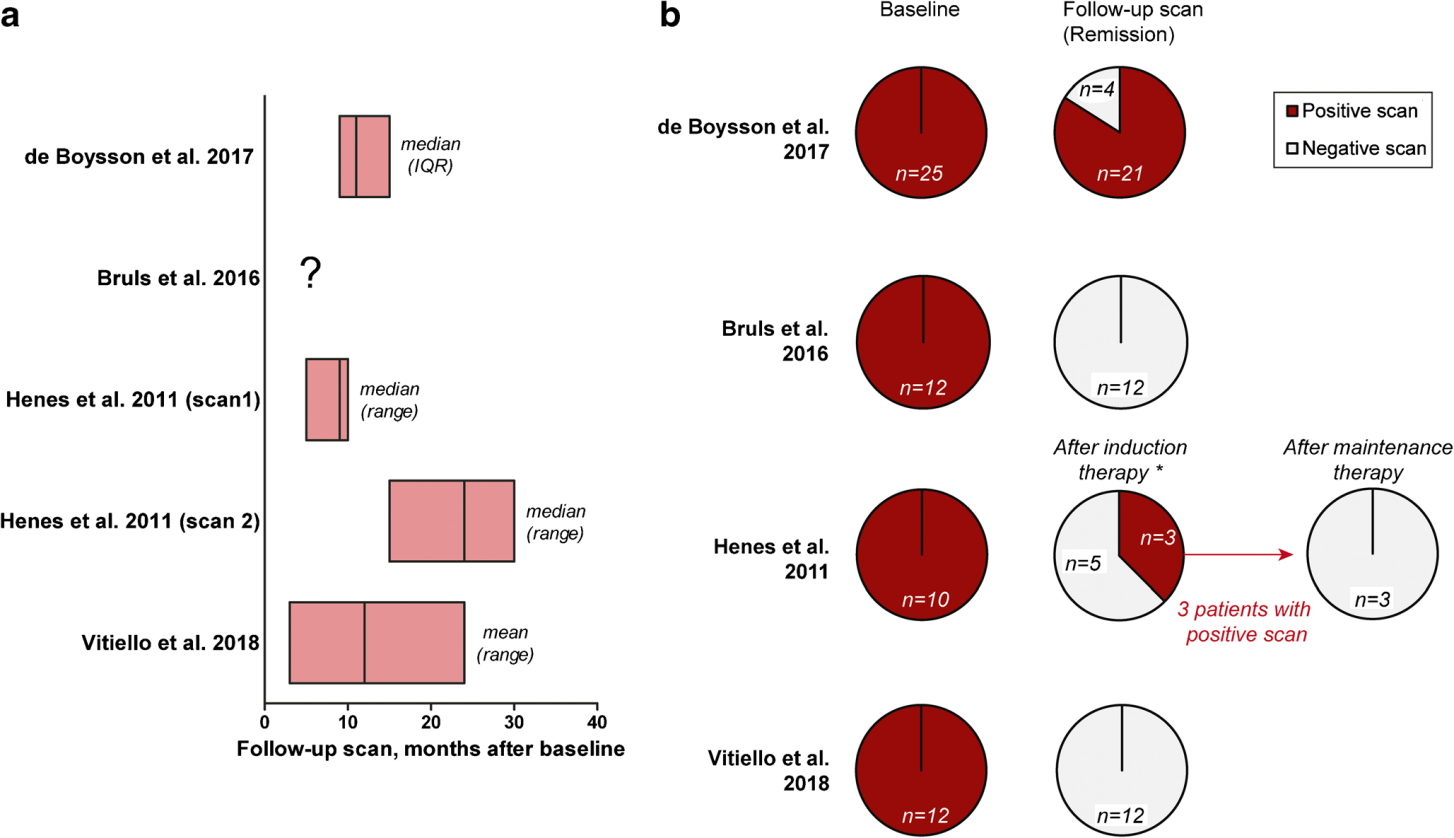
The proportion of patients with a positive [18F]FDG-PET/CT during clinical remission in longitudinal studies. Per scan data or per patient data at baseline and during serial scans were obtained, if the disease activity during the scans was clearly defined. **a** Timing of follow-up scans and **b** the number of patients with a positive scan during clinical remission in each study

Longitudinal studies indicated that the enhanced arterial [18F]FDG uptake at baseline decreases upon treatment-induced remission of LVV (Supplemental Table 2). A representative example of serial [18F]FDG-PET/CT scans in a patient with LVV is shown in Fig. 3a. In the longitudinal studies, the number of arterial segments with positive [18F]FDG uptake, composite [18F]FDG-PET/CT scores and especially target-to-background ratios (SUVartery/SUVliver) improved when patients were scanned during clinical remission (Fig. 3b and c). Two longitudinal studies indicated that [18F]FDG uptake remains high in patients with a relapsing or refractory disease on treatment (Supplemental Table 2) [19, 24]. Three studies reported complete normalization of the scans during long-term follow-up, whereas few scans became normal during remission in another study (Fig. 4). One longitudinal study investigated early [18F]FDG-PET/CT changes after initiation of high-dose glucocorticoid treatment [27]. This study showed that pathological, arterial FDG uptake disappears in 64% of patients within 10 days after the start of treatment, whereas the scans still showed pathological FDG uptake after only 3 days of treatment. In essence, the cross-sectional studies indicated that [18F]FDG uptake is higher during clinically active disease than during clinical remission (Table 3).

### Quantitative analysis (meta-analysis)

Four longitudinal studies (*n* = 57 patients) provided sufficient data to determine how often the [18F]FDG-PET/CT remained positive during clinical remission. The pooled proportion of patients with a positive follow-up scan during clinical remission was 25.4% (95% CI 0.0 to 77.1). However, marked heterogeneity was observed in the forest plot (Supplemental Figure 2). The *I*^2^ statistic was 94.1% (95% CI 88.5–96.3) indicating that pooling of proportions is not appropriate due to high heterogeneity.

**Fig. 5:**
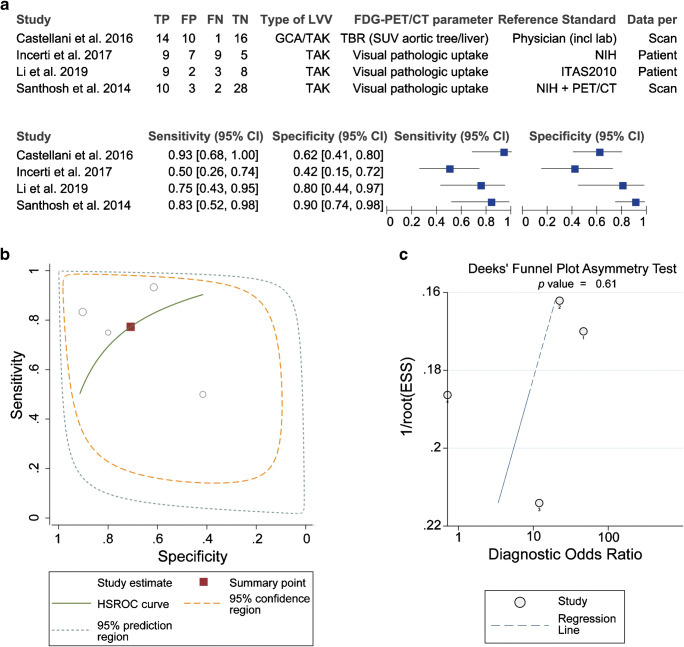
Heterogeneity and publication bias in meta-analysis of diagnostic accuracy of [18F]FDG-PET/CT during follow-up. Data were obtained from 4 cross-sectional studies in which at least 90% of patients were receiving treatment during the scan. **a** Forest plot and **b** HSROC plot of sensitivity and specificity. Pooled sensitivity was 77.3% (95%CI 56.5–89.9), and pooled specificity was 70.9% (95%CI 47.3–86.8). **c** Effective sample size (ESS) funnel plot and the associated regression test of asymmetry. A *p* value < 0.10 was considered evidence of asymmetry and potential publication bias

Four cross-sectional studies (*n* = 111 patients with 136 scans) allowed to evaluate the ability of [18F]FDG-PET/CT to distinguish the active disease from clinical remission in patients on treatment. [18F]FDG-PET/CT showed a moderate diagnostic accuracy for detecting active disease with a pooled sensitivity of 77% (95%CI 57–90%) and specificity of 71% (95%CI 47–87%) according to the bivariate model (Table 4). Substantial between-study heterogeneity was observed in the forest and HSROC plots (Fig. 5a and b). Funnel plot analysis was not suggestive of publication bias (Fig. 5c). A threshold effect did explain the heterogeneity in the studies since meta-analysis with a univariate model (Supplemental Table 3) provided similar estimates of sensitivity and specificity as the bivariate model, and no positive correlation was found between the logit of sensitivity and logit of 1 specificity (Spearman correlation coefficient − 0.40, *p* value = 0.600).

**Table 4:** Diagnostic accuracy of [18F]FDG-PET/CT for discrimination between active disease and remission during follow-up of large vessel vasculitis

No. of scans (no. scans during active disease)	Sensitivity (95% CI)	Specificity (95% CI)	Diagnostic odds ratio (95% CI)	Positive likelihood ratio (95% CI)	Negative likelihood ratio (95% CI)
136 (57)	77.3% (56.5–89.9)	70.9% (47.3–86.8)	8.27 (1.55–44.04)	2.65 (1.16–6.08)	0.32 (0.13–0.80)

Summary estimates of sensitivity, specificity, diagnostic odds ratio, positive likelihood ratio and negative likelihood ratio were determined with hierarchical logistic regression modelling (bivariate model). Data were obtained from 4 cross-sectional studies (136 scans from 111 patients) in which at least 90% of scans were performed whilst the patients were on treatment. *95% CI*, 95% confidence interval

## Discussion

### Main findings

The current systematic review and meta-analysis provide a comprehensive overview on the value of [18F]FDG-PET/CT for treatment monitoring in patients with LVV. The majority of studies indicates that arterial [18F]FDG uptake improves upon clinical remission in patients treated for LVV. It remains to be elucidated, however, to what extent [18F]FDG-PET/CT completely normalizes during clinical remission. Overall, [18F]FDG-PET/CT has moderate diagnostic accuracy to discriminate between patients with active disease and those in clinical remission. Differences in technical aspects and interpretation of [18F]FDG-PET/CT, as well as clinical differences among the included patients, may have contributed to marked between-study heterogeneity.

Current data indicate that [18F]FDG-PET/CT may aid in monitoring treatment response in patients with LVV, but its findings need to be interpreted in the context of other clinical findings. Careful evaluation of symptoms and laboratory markers remains a critical step in the assessment of disease activity. Due to its moderate diagnostic accuracy in patients on treatment (sensitivity 77%, specificity 71%), a [18F]FDG-PET/CT scan by itself cannot rule in or rule out disease activity. Prior recommendations on imaging in LVV have recognized the potential role of FDG-PET/CT for monitoring treatment response [9]. Despite the paucity of evidence, other imaging methods such as ultrasonography, MRA and CTA are also often applied to monitor treatment in LVV [12, 13]. Although [18F]FDG-PET/CT has various drawbacks including high cost and radiation exposure, it has several advantages. [18F]FDG-PET/CT is inherently a whole-body imaging method and allows for a comprehensive evaluation of all relevant large arteries in a single scan. Even temporal arteries might be evaluated due to improved resolution on newer camera systems [40, 41]. [18F]FDG-PET/CT also allows evaluation of concomitant PMR in patients with GCA [42]. Contrast-induced nephropathy is not an issue for [18F]FDG-PET/CT as no contrast is needed. Since [18F]FDG-PET/CT detects metabolic activity in the arterial wall, it could also provide complementary information to other imaging methods. For instance, persistence or gradually worsening of arterial wall thickening in the absence of [18F]FDG uptake might suggests ‘burnt out fibrotic disease’ [43]. Further studies are needed to firmly establish a role for [18F]FDG-PET/CT and other imaging methods in the management of LVV.

Factors related to [18F]FDG-PET/CT scanning and interpretation might have contributed to the between-study heterogeneity as observed in the current analysis. Differences in methodological aspects of [18F]FDG-PET/CT scan (e.g. administered activity, time interval between [18F]FDG injection and camera acquisition, scan systems and reconstruction algorithms) could lead to such heterogeneity. Scans were partly performed with a [18F]FDG-PET system in one study, which precluded inclusion in our meta-analysis [34]. Moreover, variation in scoring systems was observed across the included studies. Most included studies applied a visual uptake scoring system, with or without a semi-quantitative parameter (i.e. SUVmax). In several studies, visual scores at different arterial regions were combined into a composite [18F]FDG-PET/CT score (e.g. PETVAS), but the scan coverage and the examined arterial regions differed across the studies. Furthermore, visual grading systems used either the liver activity or blood pool activity as the reference background. The definition of [18F]FDG positivity on a visual scale as well as the optimal SUV cutoff value differed substantially between the studies and was even not reported in 29% of studies. It remains questionable which reference background is most reliable for treatment monitoring in LVV, given the increased [18F]FDG uptake by the liver due to high-dose glucocorticoids [44], and the higher [18F]FDG blood activity in patients with renal failure. This highlights the need for a standardized scoring system for LVV activity on [18F]FDG-PET/CT in addition to standardization of the scanning protocol itself. Importantly, procedural recommendations for [18F]FDG-PET/CT imaging in LVV have recently been reported [14]. The scarce data using [18F]FDG-PET/MRI in this setting does not allow any further comment on the use of MRI instead of CT, but the combination of [18F]FDG-PET and MRA may be of interest in the future.

Study heterogeneity could be further explained by patients’ characteristics in the studies. First, the number of patients on treatment during the initial scan varied among the studies. Although most cross-sectional studies only contained patients on treatment, longitudinal studies showed substantial variation in the disease stage of the included patients (i.e. newly diagnosed, relapsing and/or refractory LVV). Second, treatment differed among the studies. Marked differences existed in both glucocorticoid dosages and use of DMARDs. This could be explained by the predominant patient population in the studies (i.e. GCA versus TAK, new-onset disease versus relapsing or refractory disease) as well as local hospital preferences. Future studies should compare the effect of glucocorticoid dosage and specific DMARDs on vascular [18F]FDG uptake in patients with LVV. Third, the timing of follow-up scans differed among the studies. Follow-up scans could be performed several months or even years after treatment. It would be interesting to know the disease course preceding the follow-up scans since it might be relevant if LVV is in clinical remission for a few weeks as compared to a few months or years.

### Limitations

Our study has several limitations. The number of patients in the systematic review and especially meta-analysis was relatively small. Various types of bias may have influenced the study findings. The decision to perform [18F]FDG-PET/CT could have introduced selection bias, e.g. for patients with a refractory or relapsing disease course, who perhaps may show more persistent arterial [18F]FDG during clinical remission. In some studies, [18F]FDG-PET/CT findings were incorporated into the assessment of disease activity. This could lead to overestimation of its ability to evaluate disease activity. Another limitation was the assessment of disease activity by instruments such as the NIH criteria, ITAS2010 and BVAS in part of studies. These instruments have not been thoroughly validated for LVV and their ability to evaluate disease activity remains uncertain [12, 39]. Our study highlights a need for large, prospective studies with serial [18F]FDG-PET/CT scans at fixed time points during clinical remission in addition to scans performed at the suspicion of clinical relapse. A rigorous clinical definition of disease activity is required in the conduct of these studies as recognized by recent recommendations on the management of LVV [45].

## Conclusion

This systematic review and meta-analysis suggest that [18F]FDG-PET/CT may aid in the assessment of disease activity in patients with LVV. FDG uptake decreases during clinical remission, but it remains unclear to what extent the arterial wall [18F]FDG uptake normalizes. [18F]FDG-PET/CT has moderate accuracy to distinguish the active disease from remission in patients on treatment. Therefore, [18F]FDG-PET/CT findings should be interpreted in the context of clinical and biochemical findings. This study also highlights the relevance of procedural recommendations for [18F]FDG-PET/CT in LVV.

## Supplementary information

ESM 1 (DOCX 469 kb)
