# Supplementary material for: Diagnostic value of [18F]FDG-PET/CT for treatment monitoring in large vessel vasculitis: a systematic review and meta-analysis
Source: Eur J Nucl Med Mol Imaging. 2021 May 3;48(12):3886–902. doi: 10.1007/s00259-021-05362-8 (PMC8484162; doi:10.1007/s00259-021-05362-8)
Supplement: Supplementary file 1 — (DOCX 469 kb) [file 259_2021_5362_MOESM1_ESM.docx]

**Supplemental Table 1. Search strategy in Pubmed/MEDLINE and the Cochrane Library database.**

*Date of last search: October 21, 2020.*

| **Database** | **Search** |
| --- | --- |
| **Pubmed / MEDLINE** | (large vessel vasculit* [tiab] or "giant cell arteritis"[mesh] or giant cell arteritis [tiab] or temporal arteritis [tiab] or horton [tiab] or "Takayasu Arteritis"[Mesh] or Takayasu [tiab] or "Aortitis"[Mesh] or aortitis [tiab])  and  ("fluorodeoxyglucose f18"[mesh] or fluorodeoxyglucose [tiab] or fdg [tiab] or "positron-emission tomography"[mesh] or "positron emission tomography computed tomography"[mesh] or positron emission tomograph* [tiab] or positron-emission tomograph* [tiab] or pet [tiab])  and  (active* [tiab] OR monitor* [tiab] OR follow-up [tiab] or followup [tiab] or therap* [tiab] or treatment [tiab] or response [tiab] or relaps* [tiab] or refract* [tiab] or surveillance [tiab])  LIMITS: English Language |
| **Cochrane Library** | "large vessel vasculitis" OR "giant cell arteritis" OR "temporal arteritis" OR "horton" OR "Takayasu arteritis" OR "aortitis" in Title Abstract Keyword  AND  "fluorodeoxyglucose" OR "fdg" OR "positron-emission tomography" OR "positron emission tomography" OR "positron emission tomography computed tomography" OR "pet" in Title Abstract Keyword |

**Supplemental Table 2. Main findings in longitudinal studies on [18F]FDG-PET/CT for monitoring of treatment response.** Glucocorticoid treatment was used orally unless stated otherwise. AZA = azathioprine. BVAS = Birmingham Vasculitis Activity Score. CYC = cyclophosphamide. DMARD = disease-modifying antirheumatic drug. GC = glucocorticoid. GCA = giant cell arteritis. IFX = infliximab. IQR = interquartile range. IV = intravenous. MMF = mycophenolate (mofetil). MTX =methotrexate. n = number of patients (unless stated otherwise). NIH = National Institute of Health. SD = standard deviation. TAK = Takayasu arteritis. TBR = target to background ratio. TCZ = tocilizumab.

| **Study** | **No. of patients and disease stage** | **Disease duration since diagnosis** | **Assessment of disease activity** | **Treatment** | **FDG uptake at baseline and repeat scan during therapy** |
| --- | --- | --- | --- | --- | --- |
| Banerjee et al. 2020 ^a^ | TOTAL (n=52)  GCA (n=31)  TAK (n=21)  *Remission*  *Relapse*  *Possibly newly-diagnosed*  *Possibly refractory* | Median 1.4 years (IQR 0.6-2.7) for GCA  Median 4.8 years (IQR 1.4-14.9) for TAK | Physician global assessment on scale of 0 (remission) to 10 (very active diseases) | Longitudinal scans following increase of treatment (n=36 scans)   - Increase of GC and other immunosuppressive treatment (n=14 scans) - Increase of GC treatment only (n=4 scans) - Increase of non-GC immunosuppressive treatment only (n=18 scans)   Treatment change: change in prednisone dose by ≥ 5 mg; or either an addition or 50% dose change of a immunosuppressive treatment | Serial scans with ~6m interval following increase of treatment (n=36 scans)   - Baseline scan: PETVAS, median 23.5 (range not provided); number of scans consistent with vasculitis (global assessment), 30/36 scans; *clinically active disease during 25/36 scans* - Repeat scan: PETVAS, median 18.0 (range not provided); number of scans consistent with vasculitis (global assessment), 24/36 scans; *clinically active disease during 14/36 scans*   Serial scans with ~6m interval following unchanged treatment (n=30 scans)   - Baseline scan: PETVAS median 21.0 (range not provided); *clinical disease activity during scan unclear (median physician global assessment 0, range not provided)* - Repeat scan: PETVAS, median 21.0 (range not provided); *clinical disease activity during scan unclear (median physician global assessment 0, range not provided)*   Serial scans with ~6m interval following decrease of treatment (n=21 scans)   - Baseline scan: PETVAS median 16.0 (range not provided); *clinical disease activity during scan unclear (median physician global assessment 0, range not provided)* - Repeat scan: PETVAS, median 20.0 (range not provided); *clinical disease activity during scan unclear (median physician global assessment 0, range not provided)*   Serial scans with ~6m interval following start of TCZ (n=17 GCA patients)   - Baseline scan: PETVAS median 25.0 (range not provided); scans consistent with vasculitis (global assessment), 17/17 patients; *clinically active disease during scan in at least 10/17 patients* - Repeat scan: PETVAS median 21.5 (range not provided); scans consistent with vasculitis (global assessment), 14/17 patients; *clinically active disease during scan in 3/17 patients*   Serial scans with ~6m interval following start of IFX (n=7 TAK patients)   - Baseline scan: PETVAS, median 21.0; scans consistent with vasculitis (global assessment), 7/7 patients; *clinically active disease in 7/7 patients* - Repeat scan: PETVAS, median 16.0; scans consistent with vasculitis (global assessment), 5/7 patients; *clinically active disease in 5/7 patients* |
| Bruls et al. 2016 | TOTAL (n=12)  GCA (n=4)  TAK (n=2)  Idiopathic aortitis (n=6)  *Newly-diagnosed* | NA | Unclear (‘clinical improvement’) | Corticosteroids, azathioprine or methotrexate; aortic surgery § | Baseline scan during *clinically active disease in all patients* (n=12)   - Positive FDG uptake (i.e. ‘any increased focal uptake compared with background’ according to visual analysis) in at least one region of the aorta in all patients   Repeat scan following *clinical improvement* with immunosuppressive treatment and/or aortic surgery (n=12)   - Positive FDG uptake in at least one region of the aorta in none of the patients |
| Castellani et al. 2016 ^b^ | TOTAL (n=12)  GCA (n=9)  TAK (n=3)  *Newly-diagnosed* | NA | Assessment of clinical course (clinical and laboratory data, response to GC treatment); no standardized criteria. | GC treatment or immunosuppressants | Baseline scan during *clinically active disease in all patients* (n=12 scans)   - FDG uptake in supra-aortic branches, median grade 3 (range 0-3) †; in thoracic aorta, median grade 2 (range 0-3), in abdominal aorta, median grade 2.5 (range 0-3); in iliofemoral arteries, median grade 1 (range 0-3); total visual score of 11 regions in aortic tree, median 26 (range 0-33) - TBR (SUVmean artery/SUVmean liver) in supra-aortic branches, mean 0.78 (SD 0.16); in thoracic aorta, mean 0.96 (SD 0.14); in abdominal aorta, mean 1.09 (SD 0.21); in iliofemoral arteries, mean 0.70 (SD 0.17); in entire aortic tree, mean 0.84 (SD 0.14)   Repeat scan during *relapsing or refractory disease* (n=9 scans)   - FDG uptake in supra-aortic branches, median grade 1 (range 0-3); in thoracic aorta, median grade 2 (range 1-3); in abdominal aorta, median grade 2 (range 1-3), in iliofemoral arteries, median grade 1 (range 0-2); total visual score of 11 regions in aortic tree, median 17 (range 4-30) - TBR (SUVmean artery/SUVmean liver) in supra-aortic branches, mean 0.76 (SD 0.14); in thoracic aorta, mean 0.93 (SD 0.12); in abdominal aorta, mean 0.98 (SD 0.15); in iliofemoral arteries, mean 0.66 (SD 0.09); in entire aortic tree, mean 0.82 (SD 0.10)   Repeat scan during *complete remission* (n=20 scans)   - FDG uptake in supra-aortic branches, median grade 0 (range 0-2); in thoracic aorta, median grade 1 (range 0-2); in abdominal aorta, median grade 0.5 (range 0-3); in iliofemoral arteries, median grade 0 (range 0-1); total visual score of 11 regions in aortic tree, median 6.5 (range 0-21) - TBR (SUVmean artery/SUVmean liver) in supra-aortic branches, mean 0.63 (SD 0.10); in thoracic aorta, mean 0.79 (SD 0.11); in abdominal aorta, mean 0.82 (SD 0.19); in iliofemoral arteries, mean 0.54 (SD 0.09); in entire aortic tree, mean 0.67 (SD 0.09) |
| de Boysson et al. 2017 | GCA (n=25)  *Newly-diagnosed* | NA | Controlled disease = absence of symptoms and inflammatory markers after 3 months of GC treatment  Relapse =  re-occurrence of symptoms or re-increase of inflammatory markers without any other cause than GCA requiring increase of GC treatment | GC treatment   - Baseline (n=25), GC median dose 55 mg (IQR 40-70) - Second scan (n=25), GC median dose 10 mg (IQR 5-13); 22/25 patients on GC treatment - Third scan (n=7), median dose unclear | Baseline scan during *clinically active disease in all patients* (n=25)   - Vascular FDG uptake grade 3 † in all patients - Number of arterial regions with vascular FDG uptake grade 3, median 4 (IQR 2-5)   Second scan during *controlled disease* at median 11 months (IQR 9-15) after baseline (n=25)   - No arterial regions with vascular FDG uptake grade 3 in 4/25 patients - Less arterial regions with FDG uptake grade 3 in 8/25 patients - Unchanged number of arterial regions with FDG uptake in 10/25 patients - More arterial regions with FDG uptake grade 3 in 3/25 patients - Number of arterial regions with vascular FDG uptake grade 3, median 2 (IQR 1-4)   Third scan during *controlled disease* at median 15 months (IQR 10-24) after baseline (n=7)   - Negative third scan after negative second scan (n=1) - Negative third scan after unchanged second scan (n=1) - Less arterial regions with FDG uptake grade 3 after prior decrease on second scan (n=1) - Less arterial regions with FDG uptake grade 3 after initially unchanged second scan (n=1) - Less arterial regions with FDG uptake grade 3 after initial increase of arterial regions with vascular FDG uptake grade 3 on second scan (n=1) - Unchanged number of arterial regions with FDG uptake grade 3 after unchanged second scan (n=2) |
| Henes et al. 2011 | TOTAL (n=10)  GCA (n=6)  TAK (n=4)  *Refractory*  *Organ-threatening* | Median 7 months (range 0-36) | BVAS | Induction therapy by CYC IV pulses (n=10) with 1 patient eventually switching to oral cyclophosphamide, in combination with glucocorticoids:   - 1000 mg IV pulses followed by 1 mg/kg (n=2) - 1 mg/kg (n=5) - < 10 mg (n=2)   Maintenance therapy by: AZA (n=7), MTX (n=2) , MMF (n=1) | Baseline scan *with BVAS 5-13* (n=10)   - At least one arterial region with vascular FDG uptake grade ≥2 † in all patients - Number of arterial regions with vascular FDG uptake grade ≥2, median 4 (range 1-7)   Repeat scan following completion of cyclophosphamide induction at 5-10 months after baseline (n=10)   - No arterial region with vascular FDG uptake grade ≥2 in 5 out of 8 patients *in remission* (*with BVAS 0*); decrease of vascular FDG uptake in some regions, but similar uptake in other regions in the other 3 patients *in remission* - Decrease of vascular FDG uptake in some regions, but similar uptake in other regions in 2 patients *with active disease* (*with BVAS 3 and 5*)   Repeat scan following additional maintenance therapy at 12-30 months after baseline in patients with positive scan after induction therapy (n=4; the fifth patients lost to follow-up):   - No arterial region with vascular FDG uptake grade ≥2 in all 4 patients *in remission* (*with BVAS 0*) |
| Lee et al. 2012 ^b^ | TAK (n=13)  *Disease stage unclear (Clinically active (n=9) or inactive with vascular FDG uptake grade ≥ 1 † (n=4))* | Median 11 months (range 1-348) § | NIH criteria | Prednisolone (20-30 mg) only (n=2) or prednisolone (20-60 mg) in combination with methotrexate (n=11) | *Clinically active disease* at baseline becoming *clinically inactive* during follow-up (n=8):   - Baseline scan: number of arteries with vascular FDG uptake grade ≥ 2 †, median 8.5 (range 1-11); TBR (SUVmax artery/SUVmax liver), median 1.24 (range 0.96-2.36) - Repeat scan at median 3.5 (range 1-8) months after baseline: number of arteries with vascular FDG uptake grade ≥ 2, median 2.5 (range 0-6); TBR (SUVmax artery/SUVmax liver), median 0.76 (range 0.60-1.00)   *Clinically active disease* at baseline remaining *clinically active* during follow-up(n=1):   - Baseline scan: number of arteries with vascular FDG uptake grade ≥ 2, 5; TBR (SUVmax artery/SUVmax liver), 0.90 - Repeat scan at 10 months after baseline: number of arteries with vascular FDG uptake grade ≥ 2, 3; TBR (SUVmax artery/SUVmax liver), 0.98   *Clinically inactive disease* remaining *clinically inactive* (n=3):   - Baseline scan: number of arteries with vascular FDG uptake grade ≥ 2, median 0 (range 0-1); TBR (SUVmax artery/SUVmax liver), median 0.82 (range 0.73-0.83) - Repeat scan at 3-6 months after baseline: number of arteries with vascular FDG uptake grade ≥ 2, 0 (range 0-1); TBR (SUVmax artery/SUVmax liver), median 0.65 (range 0.63-0.75)   *Clinically inactive disease* becoming *clinically active* (n=1)   - Baseline scan: number of arteries with vascular FDG uptake grade ≥ 2, 4; TBR (SUVmax artery/SUVmax liver), 0.71 - Repeat scan at 10 months after baseline: number of arteries with vascular FDG uptake grade ≥ 2, 4; TBR (SUVmax artery/SUVmax liver), 1.12 |
| Martínez-Rodríguez et al. 2018 ^b,c^ | TOTAL (n=37)  Aortic involvement with idiopathic LVV (n=12), GCA (n=21) or other autoimmune disease (n=4)  *Newly-diagnosed*  *Refractory*  *Relapse* | 23 patients on long-term treatment at baseline; mean 3.6 years (SD 3.0) | Clinical improvement = improvement of symptoms related to underlying disease as determined by treating physician | Treatment increased/initiated after baseline scan (n=33)   - Glucocorticoids (n=27) - Methotrexate (n=19) - Tocilizumab (n=5)   No treatment increased/initiated after baseline scan (n=4) | Baseline scan during *clinically active disease* and second scan following *clinical improvement* (n=21)   - TBR (SUVmax aorta/SUVmax blood pool), mean 1.8 (SD 0.6) at baseline - TBR (SUVmax aorta/SUVmax blood pool), mean 1.5 (SD 0.3) at second scan   Baseline scan during *clinically active disease* and second scan following *no clinical improvement* (n=16)   - TBR (SUVmax aorta/SUVmax blood pool), mean 1.6 (SD 0.3) at baseline - TBR (SUVmax aorta/SUVmax blood pool), mean 1.5 (SD 0.3) at second scan   Repeat scans were performed at mean 7.5 months (SD 2.9; range 3-12) after baseline (n=37) |
| Nielsen et al. 2018 ^c^ | GCA (n=24)  *Newly-diagnosed* | NA | Patients’ global assessment of disease activity (0-10 points) and CRP | Prednisolone 60 mg for 3 days (n=10) or 10 days (n=14) after baseline | Baseline scan during *clinically active disease in all patients* (n=24); *patient’s global assessment, median 8 (IQR 5-10)*   - Vascular FDG uptake grade 3 (i.e. more than liver) in 24/24 patients - Number of arterial segments with grade 3 uptake, either median 4 (IQR 2-5) or 5 (IQR 4-5) in those with second scan at 3 days or 10 days after baseline, respectively   Repeat scan at 3 days after baseline (n=10) *with CRP decreased compared to baseline*   - Vascular FDG uptake grade 3 in 10/10 patients - Number of arterial segments with grade 3 uptake, median 3 (interquartile range 1-4) - Decrease of TBR (SUVmax artery/SUVmean venous) by 10-15% from baseline in most arterial segments   Repeat scan at 10 days after baseline (n=14); *with CRP decreased compared to baseline; patient’s global assessment, median 3.5 (IQR 3-5).*   - Vascular FDG uptake grade 3 in 5/14 patients - Median number of arterial segments with grade 3 uptake 0 (interquartile range 0-2 - Decrease of TBR (SUVmax artery/SUVmean venous) by 30-40% from baseline in most arterial segments |
| Park et al. 2018 ^a,b^ | TAK (n=11)  *Newly-diagnosed*  *Relapse* | Mean 4.4 years (SD 5.2) | Complete remission = resolution of symptoms, normalization of ESR/CRP, decrease of SUVmax on [18F]FDG-PET/CT  Partial remission = two of three remission criteria fulfilled  Therapy failure = less than two remission criteria fulfilled | IFX (n=11) with concomitant stable dose of:   - prednisolone (n=9), median dose 5 mg (range unclear) - MTX (n=8), AZA (n=2) or hydroxychloroquine (n=1) | Baseline scan during *clinically active disease in all patients* (n=11)   - Vascular FDG uptake grade ≥ 2 † in 11/11 patients - SUVmax, median 3.50 (IQR 3.10-3.84) - TBR (SUVmax artery/SUVmean vein), median 1.34 (IQR 1.13-1.95) - TBR (SUVmax artery/SUVmean liver), median 2.38 (IQR 1.47-3.05) - PETVAS, median 12.0 (IQR 11.0-15.5)   Repeat scan at 30 weeks after baseline during *complete remission* (n=3), *partial remission* (n=6), or *therapy failure* (n=2):   - Vascular FDG uptake grade ≥ 2, unclear how many patients - SUVmax, median 3.10 (IQR 2.49-3.27) - TBR (SUVmax artery/SUVmean vein), median 1.31 (IQR 1.05-1.45) - TBR (SUVmax artery/SUVmean liver), median 1.92 (IQR 1.51-2.18) - PETVAS, median 11.0 (IQR 8.0-12.0) |
| Regola et al. 2020 ^b^ | GCA (n=11)  *Newly-diagnosed*  *Relapse* | Median 6 months (10-90^th^ percentile 1-35) § | Prolonged remission = absence of symptoms and signs of active vasculitis with normalization of acute phase reactants and no evidence for progressive vessel narrowing or dilatation during ≥ 6 months  Relapse = recurrence of symptoms/signs of GCA and/or worsening of radiological imaging | TCZ + GC treatment (median dose 50 mg (10-90 percentile 25-63 mg) in all patients (n=32); additional IV methylprednisolone pulses in 3 patients; concomitant use of conventional DMARD in 10 patients § | Scans at baseline and after 6 months of treatment available (n=5 patients);   - Baseline during *clinically active disease in all patients*: PETVAS, median 19.0 (10-90^th^ percentile 9.6-22.6); TBR (SUVmax Vessel/SUVmax liver) median 1.61 (10-90 percentile 1.21-2.02) - Repeat scan with *most patients in prolonged remission* (22/25 of all patients with 6m scan in prolonged remisison §): PETVAS, median 7.0 (10-90^th^ percentile 3.2-8.0); TBR (SUVmax Vessel/SUVmax liver) median 0.86 (10-90 percentile 0.74-0.94)   Scans at baseline and after 12 months of treatment available (n=6 other patients)   - Baseline scan during *clinically active disease in all patients*: PETVAS, median 25.0 (10-90^th^ percentile 14.5 to 27); TBR (SUVmax Vessel/SUVmax liver) median 1.55 (10-90^th^ percentile 1.40-1.78) - Repeat scan during *prolonged remission in all patients*: PETVAS, median 8.0 (10-90^th^ percentile 3.5-14.5); TBR (SUVmax Vessel/SUVmax liver), median 0.88 (10-90^th^ percentile 0.78-0.93) |
| Rimland et al. 2020 ^b^ | TOTAL (n=40)  GCA (n=26)  TAK (n=14)  *Newly-diagnosed?*  *Relapse?*  *Refractory?* | Median 2.4 years (IQR 0.7-8.3) § | Physician global assessment on scale of 0 (remission) to 10 (very active diseases) | Treatment (n=112) §   - Prednisone, median dose 5 mg (IQR 0-19.4) § - Other immunosuppressant 61/112 patients § | Baseline scan during *clinically active disease* and repeat scan during *remission* (n=22-29?)   - Baseline scan: PETVAS, median 23 (IQR 16.5-25.0) - Repeat scan after median 8 months (IQR 6-15): PETVAS, median 17.5 (IQR 12.0-21.3)   Baseline scan during *remission* and repeat scan during *clinically active disease* (n=8-11?) at   - Baseline and repeat scan after median 7 months (IQR 6-10): PETVAS not significantly changed; exact values not reported |
| Sammel et al. 2020 ^a,b^ | GCA (n=15)  *Newly-diagnosed* | NA | Treating physician judgement | Glucocorticoids in all patients  Part of patients also received MTX or sirukumab | Baseline scan during *clinically active disease in all patients* (n=15)   - Total vascular score, median 14 (IQR 4-24)   Repeat scan during *unclear disease activity* after 6 months (n=15)   - Total vascular score, median 5 (IQR 0-10) |
| Vitiello et al. 2018 | GCA (n=12)  *Newly-diagnosed*  *Refractory* | Median 2.5 years (range 0-9) | Assessed by presence or absence of GCA-related symptoms and acute phase reactants | - TCZ added to treatment (n=12) at baseline - Concomitant prednisone in all patients, mean dose 27 mg (SD 13) - Concomitant MTX (n=7) | Baseline scan during *clinically active disease in all patients* (n=12)   - Vascular FDG uptake ‘consistent with vasculitis’ in 12/12 patients - SUVmean, mean 2.05 (SD 0.64)   Repeat scan during *complete remission in all patients* at mean 11.6 months (SD 8.8; range 3-24) after baseline (n=12)   - Vascular FDG uptake ‘consistent with vasculitis’ in 0/12 patients - SUVmean, mean 1.78 (SD 0.45) |

† Vascular FDG uptake grading system: 0 = no uptake, 1 = less than liver, 2 = equal to liver, 3 = more than liver.

§ including data obtained from patients without relevant data.

^a^ not included in meta-analysis: serial scan data reported for a mix of patients in remission and with active disease

^b^ not included in meta-analysis: no data on the proportion of patients in which the scan normalises or remains positive during follow-up

^c^ not included in meta-analysis: uncertainty about disease activity during serial scan

**Supplemental Table 3. Univariate analysis of sensitivity and specificity of**  **[18F]FDG-PET/CT for detection of active disease during follow-up of large vessel vasculitis.** Pooled estimates of diagnostic accuracy parameters were determined with an univariate random-effects model (DerSimonian Laird method) in MetaDiSc 1.4.95% CI = 95% confidence interval.

| **No. of scans (No. scans during active disease)** | **Sensitivity**  **(95% CI)** | **Specificity**  **(95% CI)** |
| --- | --- | --- |
| 136 (57) | 73.7 (60.3-84.5) | 72.2 (60.9-81.7) |

**Supplemental Figure 1. QUADAS-2 scoring per study.** NA = not applicable.


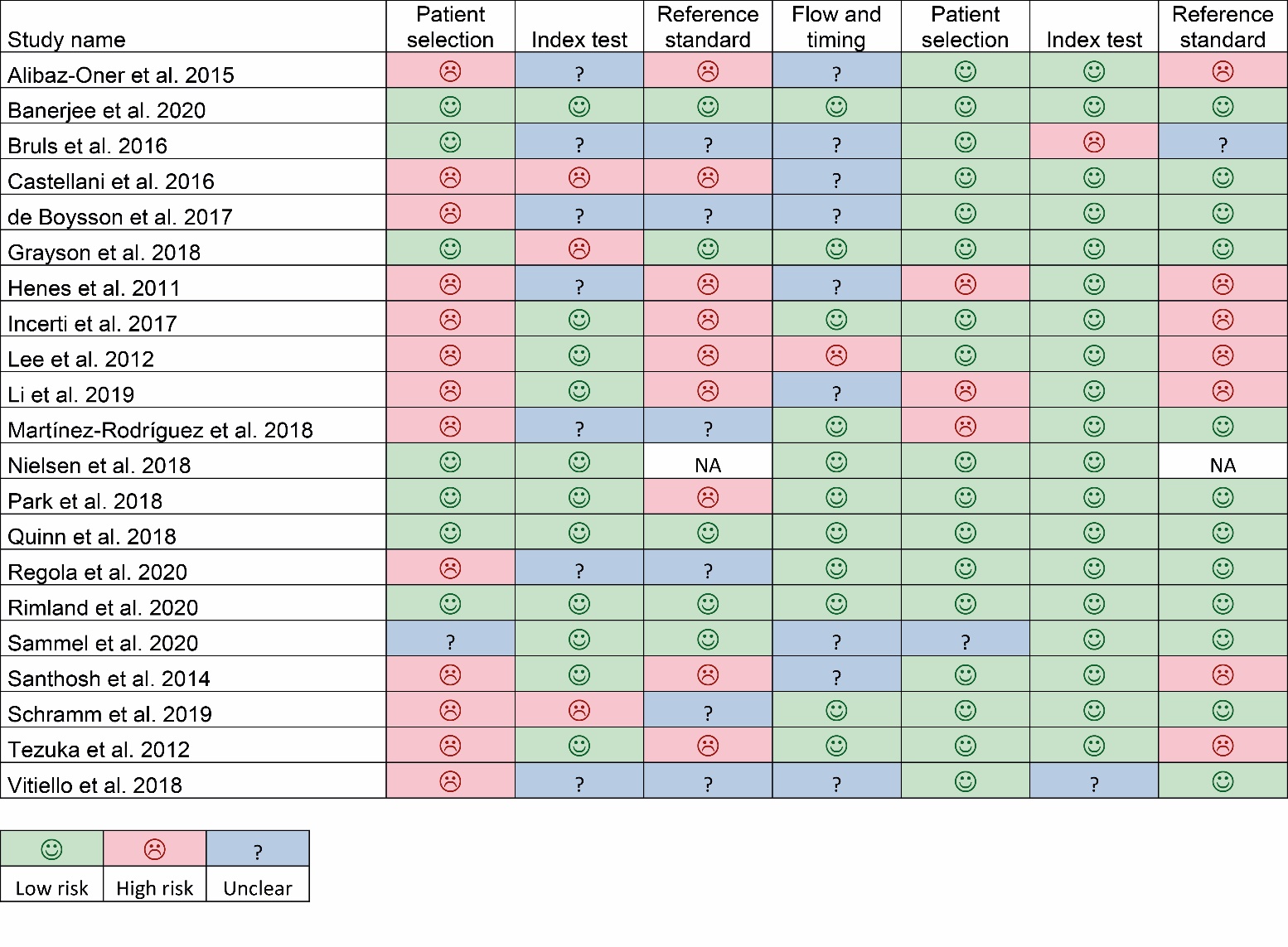


**Supplemental Figure 2. Forest plot of proportion meta-analysis.** Meta-analysis of the proportion of patients in which the [18F]FDG-PET/CT remained positive during clinical remission was performed by the Stuart-Ord (inverse double arcsine square root) method and a DerSimonian-Laird (random effects) model.
